# Ecological stoichiometry of the epiphyte community in a subtropical forest canopy

**DOI:** 10.1002/ece3.5875

**Published:** 2019-11-28

**Authors:** Jun‐Biao Huang, Wen‐Yao Liu, Su Li, Liang Song, Hua‐Zheng Lu, Xian‐Meng Shi, Xi Chen, Tao Hu, Shuai Liu, Tao Liu

**Affiliations:** ^1^ CAS Key Laboratory of Tropical Forest Ecology Xishuangbanna Tropical Botanical Garden Chinese Academy of Sciences Mengla China; ^2^ University of Chinese Academy of Sciences Beijing China; ^3^ Center of Plant Ecology Core Botanical Gardens Chinese Academy of Sciences Xishuangbanna China

**Keywords:** arboreal epiphyte, element content, functional group, nutritional strategy, phylogeny, stoichiometric ratio

## Abstract

Epiphytes in tree canopies make a considerable contribution to the species diversity, aboveground biomass, and nutrient pools in forest ecosystems. However, the nutrient status of epiphytes and their possible adaptations to nutrient deficiencies in the forest canopy remain unclear. Therefore, we analyzed the stoichiometry of five macroelements (C, N, P, K, and Ca) in four taxonomic groups (lichens, bryophytes, ferns, and spermatophytes) to investigate this issue in a subtropical montane moist evergreen broad‐leaved forest in Southwest China. We found that the interspecific variations in element concentrations and mass ratios were generally greater than the intraspecific variations. And there were significant stoichiometric differences among functional groups. Allometric relationships between N and P across the epiphyte community indicated that P might be in greater demand than N with an increase in nutrients. Although canopy nutrients were deficient, most epiphytes could still maintain high N and P concentrations and low N:P ratios. Moreover, ferns and spermatophytes allocated more limited nutrients to leaves than to stems and roots. To alleviate frequent drought stress in the forest canopy, vascular epiphytes maintained several times higher K concentrations in their leaves than in the tissues of lichens and bryophytes. Our results suggest that epiphytes may have evolved specific nutrient characteristics and adaptations, so that they can distribute in heterogeneous canopy habitats and maintain the stability of nutrient metabolism.

## Graphical abstract

Forest canopies are occupied by nearly 600 epiphytic species (including lichens, bryophytes, ferns, and spermatophytes) in a subtropical montane moist evergreen broad‐leaved forest in Southwest China. However, the nutrient status of epiphytes and their adaptations to nutrient deficiencies in the forest canopy remain unclear. In this study, we analyzed the stoichiometry of five macroelements (C, N, P, K, and Ca) of dominant epiphytes and found that stoichiometric characteristics differed across species and functional groups.

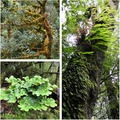

## 1 INTRODUCTION

Epiphytes form plant communities that grow on phorophytes (host trees) for physical support, but these arboreal plants do not extract any nutrients directly from the soil or the host (Benzing, 1990). Based on phylogenetic traits, epiphytes are divided into five taxonomic groups: algae, lichens, bryophytes, ferns, and spermatophytes (Coxson & Nadkarni, 1995; Deluca, Zackrisson, Nilsson, & Sellstedt, 2002; Ma, Liu, & Li, 2009; Pentecost, 1998; Pike, 1978). Epiphytes are a large part of the plant biodiversity (Gentry & Dodson, 1987; Nieder, Prosperí, & Michaloud, 2001; Wolf & Alejandro, 2003; Zotz, 2013), total canopy biomass (Coxson & Nadkarni, 1995; Nadkarni, Schaefer, Matelson, & Solano, 2004), and nutrient pools for terrestrial ecosystems (Chen, Liu, & Wang, 2009; Nadkarni, 1984; Pentecost, 1998) and play crucial roles in forest water balance and nutrient cycles (Coxson & Nadkarni, 1995; Foster, 2001; Van Stan & Pypker, 2015).

Epiphytes have no roots in the soil of the forest floor and need to efficiently access nutrients from different canopy resources (Benzing, 1990; Zotz & Hietz, 2001), such as atmospheric deposition (Clark, Nadkarni, & Gholz, 2005; Song et al., 2016; Stewart et al., 1995), stem flow and leaching from tree tissues (Wania, Hietz, & Wanek, 2002), canopy soil (Matson, Corre, & Veldkamp, 2014; Reich, Ewel, Nadkarni, Dawson, & Evans, 2003), organic matter decomposition (Hietz, Wanek, & Popp, 1999; Hietz, Wanek, Wania, & Nadkarni, 2002), atmospheric N_2_ fixation by lichens and bryophytes through their symbiotic cyanobacteria (Adams & Duggan, 2008; Asplund & Wardle, 2017; Deluca et al., 2002), and animals and the organic matter they import (Treseder, Davidson, & Ehleringer, 1995). However, the forest canopy for epiphytes has generally been considered as an extreme habitat (Benzing, 1990), where water and nutrients are limited (Benzing, 1990; Zotz & Hietz, 2001), irregularly available (Laube & Zotz, 2003), and spatially variable (Hietz et al., 2002). Therefore, the study of the nutrient status of epiphytes and their nutritional adaptations is necessary. Ecological stoichiometry from marine and terrestrial plants may provide some methods and establish some criteria for the evaluation of epiphytes.

Ecological stoichiometry reveals the balance of multiple chemical elements in ecological interactions and processes, which is also referred to as the balance of energy and materials (Elser et al., 2000; Sterner & Elser, 2002). Four aspects of ecological stoichiometry may be helpful to the stoichiometric study of epiphytes. First, the growth rate hypothesis proposes that rapidly growing organisms commonly have low biomass C:P and N:P ratios that reflect increased allocation to P‐rich ribosomal RNA (Sterner & Elser, 2002) and growth rates that correlate positively with RNA, N, and P contents (Ågren, 2004; Elser et al., 2003; Hessen, Jensen, Kyle, & Elser, 2007; Nielsen, Enríquez, Duarte, & Sand‐Jensen, 1996). Second, the argument for stoichiometric homoeostasis is that organisms have the ability to maintain a given elemental composition despite variation in the elemental composition of its environment or resource supplies (Sterner & Elser, 2002). Some degree of flexibility or low degree of homeostasis indicates that plants can change their elemental stoichiometries in response to changes in resource availability (Koerselman & Meuleman, 1996; Yu et al., 2015). Third, the threshold ratios of N and P are widely used to predict N or P limitation in plants (Güsewell, 2004; Güsewell, Koerselman, & Verhoeven, 2003; Koerselman & Meuleman, 1996). However, the indirect evidence based on the N:P ratios is not always reliable (Yan, Tian, Han, Tang, & Fang, 2017). Last, scaling relationships between nitrogen and phosphorus are widely found in different plant organs and plant functional groups (Kerkhoff, Fagan, Elser, & Enquist, 2006; Zhang et al., 2018). Leaf nitrogen is usually scaled as 2/3 or 3/4 the power of leaf phosphorus (Niklas, Owens, Reich, & Cobb, 2005; Reich et al., 2010), which can be used to predict the relative growth rates of plants (Niklas, 2006).

The ecological stoichiometry in plants can be influenced not only by environmental factors but also by species, organs, and functional types. In large‐scale studies, the elemental composition and stoichiometry of terrestrial plants are influenced by forest type, climate, and soil (Chen, Han, Tang, Tang, & Fang, 2013; Han, Fang, Reich, Woodward, & Wang, 2011; Sardans et al., 2015; Tian et al., 2018). In a European forest, the identity of tree species can explain 56.7% of the variance of the overall foliar elemental composition and stoichiometry (Sardans et al., 2015). Between plant organs, the scaling relationship of N and P differs between primarily structural organs (stems and roots) and metabolically active leaves (Kerkhoff et al., 2006). In an arid and hot grassland, plants allocate more resources to leaves than to stems for adaptations to the nutrient‐limited environment (Yan et al., 2016). The stoichiometry is different among different functional groups of terrestrial plants, including between herbaceous and woody plants (Kerkhoff et al., 2006; Tian et al., 2018), deciduous and evergreen plants (Aerts & Chapin, 1999; Güsewell, 2004), gymnosperms and angiosperms (Sardans et al., 2016), and among herbs, shrubs, and trees (Han, Fang, Guo, & Zhang, 2005). Deciduous plants generally have mineral‐rich leaves compared with those of evergreen plants (Aerts & Chapin, 1999; Chen et al., 2013; Güsewell, 2004; Han et al., 2005, 2011), and between plant functional types, the N and P contents are higher in herbs than those in woody plants (Han et al., 2005; Tian et al., 2018).

Epiphytes can survive and flourish in the forest canopy because they evolved a diversity of morphological, anatomical, and physiological adaptations (Benzing, 1990; Zotz & Hietz, 2001). Many morphological structures of epiphytes contribute to obtain and share nutrients, such as tank leaves of epiphytic bromeliads (Hietz & Wanek, 2003; Inselsbacher et al., 2007; Winkler & Zotz, 2009) and trichomes of tank leaves for nutrient uptake (Winkler & Zotz, 2010), intact rhizomes for resource sharing in epiphytic ferns (Lu et al., 2016), the velamen of aerial roots for nutrient uptake in epiphytic orchids (Zotz & Winkler, 2013), and older and leafless stems for resource storage in an epiphytic orchid (Zotz, 1999). Mycorrhizae in epiphytic orchids occur widely and increase the uptake of water and mineral nutrients (Lesica & Antibus, 1990). Moreover, the C_3_‐CAM epiphytes have higher long‐term water use efficiency for net CO_2_ uptake than that of the C_3_ epiphytes (Zotz & Winter, 1994). The resorption of nutrients in vascular epiphytes can also alleviate nutrient restrictions in the canopy (Zotz, 2004). However, the growth of epiphytes remains limited, as indirectly demonstrated by the low contents of nutrient elements (Hietz et al., 1999; Hofstede, Wolf, & Benzing, 1993; Zotz, 2004; Zotz & Richter, 2006), high foliar N:P (10.2–33.8) ratios (Lasso & Ackerman, 2013; Wanek & Zotz, 2011; Zotz, 2004), widespread P limitation (Benner & Vitousek, 2007; Cardelús & Mack, 2010; Zotz & Richter, 2006), and very slow growth rates (Laube & Zotz, 2003; Schmidt & Zotz, 2002).

Although the nutrient sources of epiphytes and their adaptations to canopy habitats were identified in previous studies (Hietz et al., 1999; Song et al., 2016; Zotz & Hietz, 2001), the nutrient status and adaptations of the entire epiphyte community remain unclear. In stoichiometric studies of terrestrial plants, K and Ca are rarely studied, although these elements may be important for epiphytes because K alleviates drought stress in plants (Sardans & Peñuelas, 2015; Sardans, Peñuelas, Coll, Vayreda, & Rivas‐Ubach, 2012), and Ca^2+^ is an intracellular secondary messenger that transmits signals of environmental changes (Bush, 1995; Lecourieux, Ranjeva, & Pugin, 2006). In this study, the C, N, P, K, and Ca contents and their ratios in the dominant epiphyte species of lichens, bryophytes, ferns, and spermatophytes were analyzed. Based on the theory of ecological stoichiometry, the objectives of this study were the following: (a) to determine the stoichiometric characteristics of epiphytes across different levels of elements, organs, species, functional groups, and communities; (b) to determine the stoichiometry of K and Ca and their roles in epiphytes; and (c) to speculate possible nutritional adaptations in epiphytes.

## 2 MATERIALS AND METHODS

### 2.1 Study site

The study was conducted in the Ailao Mountains National Nature Reserve (23°35′–24°44′N, 100°54′–101°30′E) in the central area of Yunnan Province, Southwest China. The reserve is at an altitude of 2,000–2,750 m. The annual mean air temperature is 11.3°C, with a minimum monthly mean temperature of 5.7°C in January and a maximum monthly mean temperature of 15.6°C in July, and the mean annual precipitation is 1,841 mm, with 86% falling in the rainy season from May to October. The mean annual relative humidity of the reserve is 85% (Li, Liu, & Li, 2013). Montane moist evergreen broad‐leaved forest is the predominant vegetation. The dominant tree species in the forest are *Lithocarpus xylocarpus*, *L. hancei*, *L. chintungensis*, *Schima noronhae*, *Manglietia insignis*, and *Castanopsis wattii* (Li et al., 2013; Ma et al., 2009).

The primary forest in the reserve has a high diversity of epiphytes. The branch and trunk surfaces of trees are occupied by nearly 600 epiphytic species (Li et al., 2013), including lichens (183), bryophytes (176), ferns (117), and spermatophytes (113) (Li et al., 2013, 2014; Ma et al., 2009; Xu & Liu, 2005). The dominant lichens are *Usnea florida*, *Cetrelia olivetorum*, *Everniastrum nepalense*, *Nephromopsis ornata*, and *N. pallescens*. The dominant bryophytes are *Plagiochila assamica*, *Homaliodendron flabellatum*, *H. scalpellifolium*, *Calyptothecium hookeri*, and *P. subtropica*. The dominant ferns are *Lepisorus scolopendrium*, *Polypodiodes subamoena*, *Araiostegia perdurans*, *Vittaria flexuosa*, and *Oleandra wallichii*. The dominant spermatophytes are *Agapetesm annii*, *Aeschynanthus buxifolius*, *Briggsia longifolia*, and *Cautleya gracilis*.

### 2.2 Experimental design and sampling

In the primary forest, six experimental plots (60 m × 60 m) were set up for sampling in 2014. Then, two plots were chosen to identify all epiphytes and their host trees and to investigate the distribution of epiphytes on host trees in the early rainy season. To choose the dominant epiphytes and their primary host tree species for the study, field data were combined with literature information of epiphyte diversity in this region (Li et al., 2013; Ma et al., 2009; Xu & Liu, 2005). Ultimately, twenty dominant epiphytes were selected, depending on the actual situation when sampling in the field. The twenty dominant species included six lichen species, five bryophyte species, six fern species, and three spermatophyte species (Table 1). The lichen species were chlorolichens containing green algae as their photobiont.

**Table 1:** Element concentrations and mass ratios of dominant epiphytes (mean ± *SD*)

Phylogenetic groups	Species	C (mg/g)	N (mg/g)	P (mg/g)	K (mg/g)	Ca (mg/g)	N:P	N:K	N:Ca
Lichens	*Usnea florida*	436.00 ± 2.92fg	8.80 ± 0.53f	0.46 ± 0.09d	3.17 ± 0.40fg	3.70 ± 0.50d	19.79 ± 3.48a	2.81 ± 0.37d	2.41 ± 0.32cd
	*Nephromopsis ornata*	442.67 ± 5.13efh	8.99 ± 0.71f	0.70 ± 0.18cd	3.85 ± 0.74efg	6.83 ± 2.14bcd	13.47 ± 3.44abcdf	2.41 ± 0.49ade	1.45 ± 0.56ce
	*Everniastrum cirrhatum*	452.00 ± 1.00e	10.60 ± 1.00ef	0.66 ± 0.13cd	3.22 ± 0.40fg	2.85 ± 0.31d	16.58 ± 3.67ab	3.33 ± 1.53acd	3.74 ± 0.39ab
	*Cetrelia braunsiana*	457.00 ± 7.70cjh	8.54 ± 0.82f	0.65 ± 0.05cd	3.31 ± 0.04fi	3.12 ± 0.25de	13.55 ± 0.80abcdg	2.63 ± 0.10ad	2.81 ± 0.17bde
	*Ramalina conduplicans*	436.17 ± 2.86f	8.72 ± 0.66f	0.49 ± 0.08d	1.99 ± 0.15h	1.05 ± 0.34e	18.18 ± 1.82a	4.39 ± 0.30c	8.91 ± 2.54a
	*Rimelia cetrata*	463.25 ± 1.71c	9.52 ± 1.57ef	1.39 ± 0.43bcd	3.75 ± 0.37efgh	2.65 ± 0.65cde	7.08 ± 1.15fg	2.54 ± 0.42adef	3.64 ± 0.51abcd
Bryophytes	*Plagiochila assamica*	476.30 ± 6.14b	19.86 ± 2.80c	1.76 ± 0.34b	5.20 ± 1.39e	5.39 ± 0.73c	11.59 ± 2.02abeg	4.22 ± 1.71acd	3.72 ± 0.54ab
	*Homaliodendron flabellatum*	454.65 ± 2.91de	18.60 ± 2.29c	2.20 ± 0.51ab	3.18 ± 0.72fg	8.34 ± 0.72b	8.83 ± 2.14cdf	6.20 ± 1.87ab	2.25 ± 0.38ce
	*Homaliodendron scalpellifolium*	456.50 ± 3.31d	18.62 ± 2.45c	2.14 ± 0.38a	2.56 ± 0.56g	9.04 ± 0.89b	8.90 ± 1.78cdf	7.71 ± 2.28b	2.07 ± 0.30ce
	*Calyptothecium hookeri*	456.46 ± 5.14de	20.91 ± 2.46c	1.60 ± 0.54bc	3.44 ± 0.87fgh	7.92 ± 1.11b	13.99 ± 3.88abcdf	5.41 ± 2.48abcdg	2.32 ± 1.06bc
	*Plagiochila subtropica*	458.63 ± 11.17c	19.92 ± 3.44c	1.51 ± 0.59bcd	2.49 ± 0.71ghi	9.57 ± 1.83ab	16.44 ± 6.78abcdf	9.49 ± 3.82abcd	2.37 ± 0.76bc
Ferns (leaf)	*Lepisorus scolopendrium*	439.17 ± 8.97fghij	22.92 ± 3.53bc	2.67 ± 1.00ab	34.16 ± 6.59a	7.66 ± 1.50b	9.58 ± 3.74bcdf	0.68 ± 0.12g	3.13 ± 1.21bcd
	*Haplopteris flexuosa*	450.77 ± 4.25eh	24.81 ± 2.13b	2.99 ± 0.61a	23.20 ± 3.24b	4.15 ± 0.85cd	8.63 ± 2.03df	1.09 ± 0.17f	6.18 ± 1.23a
	*Araiostegia perdurans*	462.29 ± 4.25c	20.48 ± 3.06c	1.73 ± 0.61b	18.58 ± 3.65bc	5.45 ± 0.89c	12.88 ± 3.65abcd	1.13 ± 0.21f	3.84 ± 0.83ab
	*Polypodiodes subamoena*	456.38 ± 5.91de	26.87 ± 3.55ab	2.57 ± 0.97ab	28.06 ± 5.03ab	5.03 ± 0.90cd	11.69 ± 3.90abcdf	0.98 ± 0.17f	5.45 ± 0.81a
	*Asplenium ensiforme*	429.56 ± 2.07g	25.14 ± 2.31ab	3.34 ± 0.84a	17.30 ± 1.44c	10.74 ± 0.90a	7.93 ± 1.97f	1.46 ± 0.18e	2.36 ± 0.29ce
	*Asplenium indicum*	442.44 ± 3.01fij	29.24 ± 2.48a	2.39 ± 0.50ab	13.00 ± 1.72d	8.86 ± 1.05ab	12.64 ± 2.2abe	2.27 ± 0.24ad	3.35 ± 0.56abd
Spermatophytes (leaf)	*Agapetes mannii*	522.00 ± 17.57a	11.13 ± 1.68ef	0.75 ± 0.02c	6.11 ± 1.91efgh	6.70 ± 1.91bcd	14.33 ± 2.13abce	1.81 ± 0.37adef	1.70 ± 0.50ce
	*Aeschynanthus buxifolius*	456.22 ± 19.70 bcfghi	14.12 ± 3.05de	1.42 ± 0.57bcd	19.77 ± 7.24abcdef	9.49 ± 2.21ab	9.80 ± 2.13bcdf	0.71 ± 0.19fg	1.45 ± 0.45c
	*Briggsia longifolia*	439.18 ± 2.96fij	16.27 ± 0.96d	2.01 ± 0.51ab	14.58 ± 2.21cd	8.68 ± 0.73b	8.62 ± 2.48def	1.14 ± 0.19ef	1.89 ± 0.20ce
All species		454.38 ± 19.58	17.20 ± 6.77	1.67 ± 0.87	10.55 ± 9.86	6.36 ± 2.78	12.23 ± 3.61	3.23 ± 2.78	3.25 ± 1.82
Leaf of dominant tree species in the study site			12.88 ± 1.74	0.97 ± 0.17	7.30 ± 2.25	5.40 ± 0.91	13.38 ± 1.54	(Liu, Fox, & Xu, 2002)	
Leaf of terrestrial plants in China			20.20	1.45			16.30	(Han et al., 2005)	
Leaf of global terrestrial plants			20.10	1.77			13.80	(Reich & Oleksyn, 2004)	

Note

Different letters indicate significant differences (*p* < .05).

Field sampling was conducted during the mid‐period of the rainy season (from July 15 to August 15). This sampling period was the most suitable for epiphyte growth throughout the year. In each plot, 20–30 replicate samples of each epiphytic species were collected from their primary host trees. These replicate samples were collected randomly from different tree individuals and were prepared separately by tree species. Mature and healthy plants were collected for samples. The bryophytes were mainly collected from tree trunks. The ferns and spermatophytes were collected from the trunks and primary and secondary branches on the host trees. The lichens were mainly collected from the outer branches. These samplings were completed with self‐made high‐branch scissors and ladders.

All plant samples were carefully cleaned with distilled water and oven‐dried at 70°C for 48 hr. The dried plant samples of roots, stems, and leaves from ferns and spermatophytes were prepared separately. All samples were ground to fine powder, using a small plant grinder. Some small samples from lichens and bryophytes were cut into sufficiently small pieces with scissors and then ground to powder with a mortar.

### 2.3 Chemical analyses

All powder samples were oven‐dried at 60°C for 24 hr before analysis of the nutrient concentrations. The total C and N concentrations of the plant samples were determined with an elemental analyzer (Vario MAX CN Elemental Analyzer, Elementar). Before measuring the total P, K, and Ca concentrations, plant samples were digested with HNO_3_‐HClO_4_. All samples were analyzed for P, K, and Ca with an inductively coupled plasma atomic emission spectrometer (iCAP6300, Thermo Fisher Scientific, Inc.).

### 2.4 Statistical analyses

All the element ratios were calculated on the basis of mass in this study. One‐way ANOVA and multiple comparisons were used to analyze the differences in element concentrations and mass ratios among epiphyte species. This variance analysis was also conducted to compare whether differences occurred in different organs or functional groups. To compare stoichiometric characteristics at the species level, the leaves of ferns and spermatophytes were chosen, because leaves were the photosynthetic structures of these vascular plants and were equivalent to lichen and bryophyte tissues. Before the analysis of variance, all data were subjected to tests for normality and homogeneity. The data that met the homogeneity test were analyzed with Fisher's least significant test (LSD). The data that did not pass the test for homogeneity were analyzed by the Kruskal–Wallis H test. The power function model (Y = bX^a^) was used to explore the relationships between N and P. The significance of the regression models was determined by an *F* test. A factor analysis (FA) was performed to analyze the possible relationships between epiphytes and their element stoichiometry (element concentrations and ratios). The common factors in the factor analysis were rotated by varimax. All statistical analyses were performed using the SPSS statistical software package v.19.0 (IBM Corporation).

## 3 RESULTS

### 3.1 Element concentrations and mass ratios across all species

The mean concentrations of C, N, P, K, and Ca for all the epiphytes were 454.38, 17.20, 1.67, 10.55, and 6.36 mg/g (C:N:P:K:Ca = 272:10:1:6:4), respectively (Table 1). The element concentrations and ratios varied widely among epiphyte species, with wide ranges of values for C (429.56–522.00 mg/g), N (8.54–29.42 mg/g), P (0.46–3.34 mg/g), K (1.99–34.16 mg/g), Ca (1.05–10.74 mg/g), N:P (7.08–19.79), N:K (0.68–9.49), and N:Ca (1.45–8.91). Most epiphyte species also differed significantly within the taxonomic groups (i.e., lichens, bryophytes, ferns, and spermatophytes) (*p* < .05). Only in a few cases were nutrients or their ratios not significantly different among the species within a group, such as N in lichens, N in bryophytes, P in lichens, and the N:Ca ratio in spermatophytes. The mean N, P, K, and Ca concentrations in the epiphytes were higher than those in the leaves of the host trees in this study site. However, the N and K concentrations in most lichens were lower than those in the leaves of the host trees. The mean N:P ratio of epiphytes was 12.23, which was similar to that in the leaves of the tree species in this study site or to that in terrestrial plants globally but lower than that in the leaves of terrestrial plants in China.

### 3.2 Patterns of stoichiometry across functional groups and plant organs

**Figure 1:**
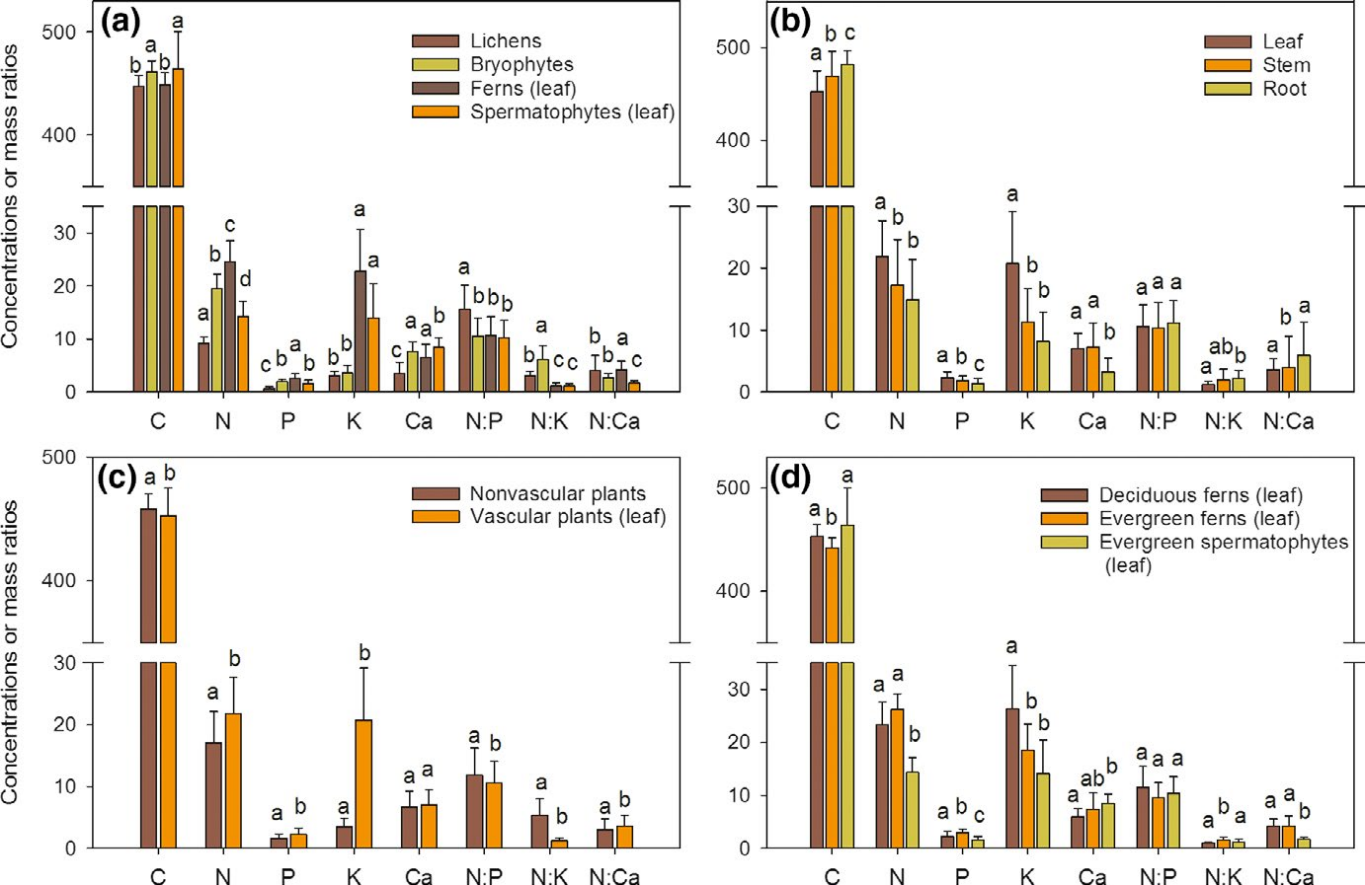
Element concentrations (mg/g) and mass ratios across different functional groups and plant organs. (a) Phylogenetic groups (lichens, bryophytes, ferns, and spermatophytes), (b) plant organs (leaf, stem, and root), (c) vascular tissue differentiation (nonvascular plants and vascular plants), and (d) leaf habits (deciduous ferns, evergreen ferns, and evergreen spermatophytes). Different lowercase letters represent significant differences (*p* < .05). Whiskers on bars denote standard deviations (*SD*)

The element concentrations and mass ratios of epiphytes were compared among phylogenetic groups (Figure 1a). The lichens and the leaves of ferns had lower C concentrations than the bryophytes and the leaves of spermatophytes. The N concentrations were significantly different among the phylogenetic groups (ferns > bryophytes>spermatophytes > lichens) (*p* < .05). The lowest and highest P concentrations were in the lichens and the leaves of ferns, respectively. The K concentrations in the leaves of ferns and spermatophytes were significantly higher than those in the lichens and bryophytes (*p* < .05). The highest Ca concentration was in the leaves of spermatophytes. The lichens had higher N:P ratios than those in the other plant groups. The order of the N:K ratio among groups was bryophytes > lichens>ferns and spermatophytes. The leaves of the spermatophytes had the lowest N:Ca ratios.

The leaves of ferns and spermatophytes had the highest N, P, and K concentrations, compared with the stems and roots (Figure 1b). The concentrations of Ca in the leaves and stems were higher than those in the roots. The difference in C among plant organs was in the order leaf < stem <root. The N:P ratio was not significantly different among plant organs. The N:K ratios in leaves were significantly lower than those in the roots (*p* < .05); however, the N:Ca ratio was not different between the organs.

The values for C, N, P, K, N:P, N:K, and N:Ca were significantly different between nonvascular plants and vascular plants (*p* < .05) (Figure 1c). Only the Ca content was not different between the two groups. The values for N, P, K, and N:Ca were higher in the vascular plants, whereas the values of C, N:P, and N:K in the nonvascular plants were higher than those in the vascular plants.

The deciduous ferns had higher K concentrations in their leaves than those in the leaves of evergreen ferns and evergreen spermatophytes (Figure 1d). The leaf P concentrations were different among the three groups and were in the order evergreen ferns > deciduous ferns > evergreen spermatophytes. No significant differences in N, Ca, and N:Ca values were found between the deciduous and evergreen ferns. The N:P ratios among the three plant groups were similar. The deciduous ferns had higher C concentrations and lower N:K ratios in their leaves than those in the leaves of evergreen ferns.

### 3.3 Scaling relationships between N and P

**Figure 2:**
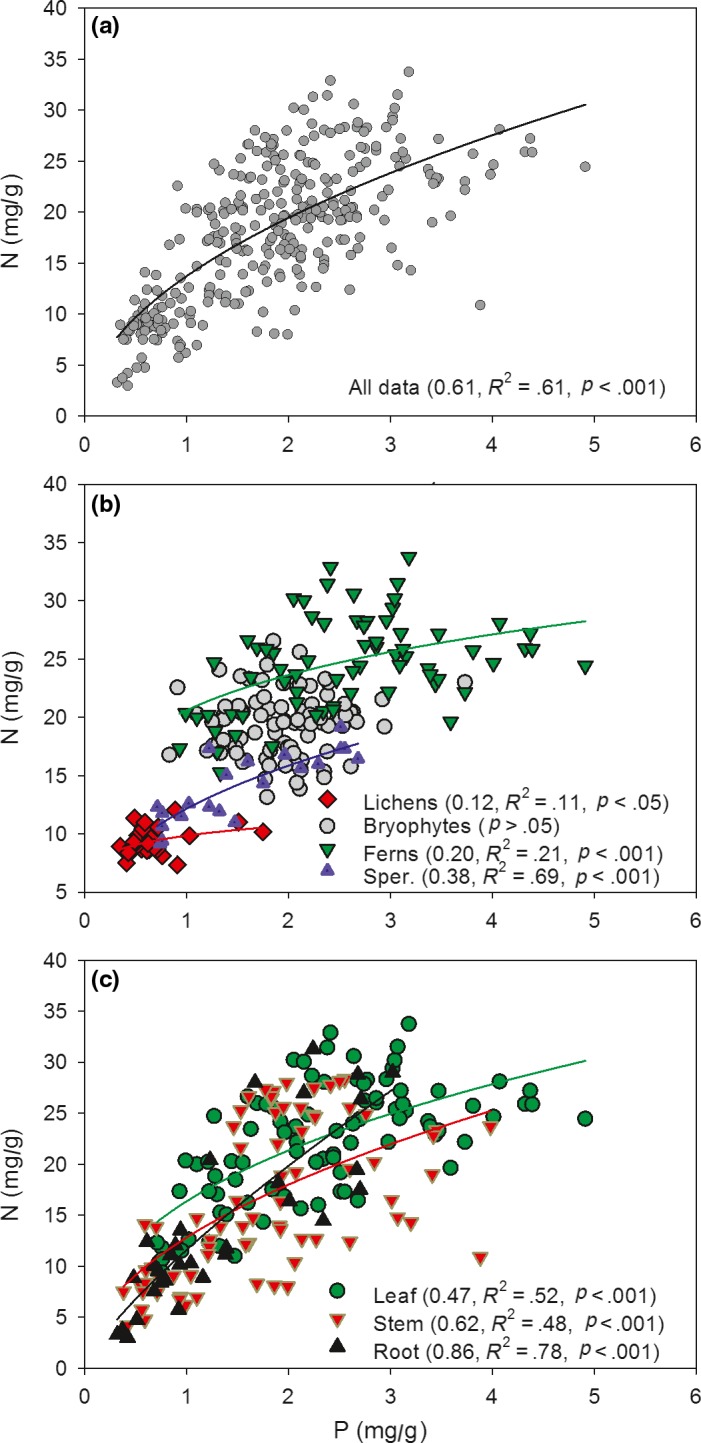
The scaling relationships between N and P concentrations fitted by N = bP^a^ in the epiphytes (a, *R* ^2^, *p*). (a) Pooled data include data from (b) and (c); (b) relationships in lichens, bryophytes, leaves of ferns, and leaves of spermatophytes; (c) relationships in the leaf, stem, and root of ferns and spermatophytes. Sper., spermatophytes

The scaling relationship between N and P was significantly positive (0.61 for the scaling exponent) in the pooled data of epiphytes (*p* < .001) (Figure 2a). However, when the phylogenetic groups were separated from the pooled data, the scaling exponents in the phylogenetic groups were less than 0.61 (Figure 2b). These scaling exponents were ranked in the following order: lichens (0.12) < leaf of ferns (0.20) < leaf of spermatophytes (0.38). The relationship between N and P in the bryophytes was not statistically significant (*p* > .05). The allometric relationships between N and P were also observed in the leaf (0.47), stem (0.62), and root (0.86) of ferns and spermatophytes (Figure 2c).

### 3.4 Relationships between epiphytes and their stoichiometric characteristics

**Figure 3:**
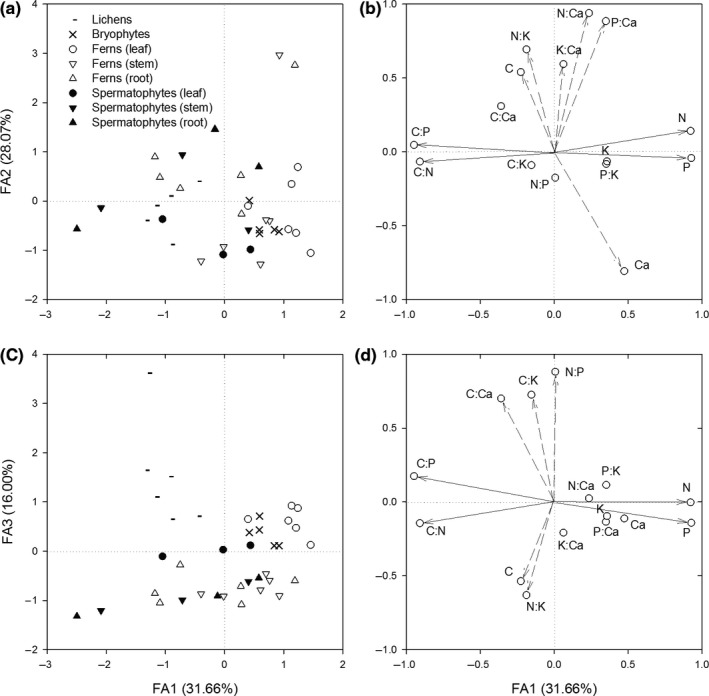
Factor analysis (FA) of epiphyte species with all the element concentrations and mass ratios. FA1, FA2, and FA3 are the first three factors and explain 75.73% of the total variance in the original variables of element concentrations and mass ratios. (a) and (c) are the distributions of epiphytes with factor scores at FA1, FA2, and FA3. (b) and (d) are the factor loadings of the original variables at FA1, FA2, and FA3. Solid arrows represent high factor loadings of the original variables at FA1. Dashed arrows represent high factor loadings of the original variables at FA2 and FA3

The relationships between epiphytes and their stoichiometry were detected by factor analysis (Figure 3). The first three factors explained 31.66%, 28.07%, and 16.00% of the total variance in the original variables. The first factor (FA1) primarily represented N, P, C:N, and C:P. The second factor (FA2) primarily represented C, N:K, K:Ca, N:Ca, P:Ca, and Ca. The third factor (FA3) primarily represented C:Ca, C:K, N:P, C, and N:K. The original variables in the same factor had high loadings, which indicated relatively high associations between them. For example, N and P in the FA1 had high positive loadings that also indicated high positive associations between the two elements in the epiphytes.

All species of lichens were distributed on the negative side of FA1, which indicated relatively low N and P concentrations and relatively high C:N and C:P ratios (Figure 3a). The maximum negative value in the FA1 was in the tuberous root of *Agapetes mannii* (spermatophyte species). The bryophytes and leaves of ferns were distributed on the positive side of FA1. In the FA2, the bryophytes and leaves of spermatophytes were distributed on the negative side. The maximum positive value in the FA2 was in the stem of *Haplopteris flexuosa* (fern species). In the FA3, the lichens, bryophytes, and leaves of spermatophytes were distributed on the positive side (Figure 3c), whereas the stems and roots were distributed on the negative side. The maximum positive value came from *Ramalina conduplicans* (lichen species), which had relatively high C:Ca, C:K, and N:P ratios but relatively low C and N:K values.

## 4 DISCUSSION

### 4.1 Ecological stoichiometry of epiphytes in the forest canopy

The epiphytes had highly variable element concentrations and mass ratios at the species level (Table 1) that were also reflected in the dispersive distribution of epiphytes on the factors (Figure 3). The high variability was most likely because of the highly heterogeneous environment and variety of nutrient sources in the forest canopy (Benzing, 1990; Hietz et al., 2002; Zotz & Hietz, 2001). Furthermore, highly variation in the element concentrations and mass ratios might also indicated different nutrient limitations or nutrient requirements across species. The N:P ratio (7.08–19.79) in the epiphytes indicated that N‐P thresholds or N‐P nutrient status might be different among epiphyte species. In terrestrial plants, the N:P ratio thresholds of 14 and 16 (Koerselman & Meuleman, 1996) or of 10 and 20 (Güsewell, 2004) are used to indicate N, P, or N‐P limitation. However, the N‐P fertilization of the tank bromeliad *Vriesea sanguinolenta* indicated that the critical foliar N:P ratio was between 10 and 12, with foliar N:P ratios > 12 indicating P limitation (or colimitation by N and P) (Wanek & Zotz, 2011). According to these thresholds, N limitation, P limitation, and N‐P colimitation might occur simultaneously in different epiphytes. However, in previous studies, the growth of vascular epiphytes was primarily P limited (Benner & Vitousek, 2007; Johansson, Olofsson, Giesler, & Palmqvist, 2011; Lasso & Ackerman, 2013; Wanek & Zotz, 2011; Zotz & Asshoff, 2010; Zotz & Richter, 2006). Although these thresholds are not always invariable, the prediction is that higher N:P ratios in epiphytes would most likely indicate P limitation, whereas lower N:P ratios would most likely indicate N constraint (Yan et al., 2017). For example, *Asplenium indicum* (N_leaf_:P_leaf_ = 7.93) might be more likely N limited than *Araiostegia perdurans* (N_leaf_:P_leaf_ = 12.88) in the fern group (Table 1).

Element concentrations and mass ratios of epiphytes differed significantly among functional groups and organs (Figure 1). Differences among functional groups are also widespread in other terrestrial plants (Güsewell, 2004; Han et al., 2005; Sardans et al., 2016; Tian et al., 2018). In this study, these differences might be caused by the large differences in morphological, anatomical, and physiological traits among lichens, bryophytes, ferns, and spermatophytes that led to differences in nutrient uptake, nutrient metabolism, and nutrient retention (Aerts & Chapin, 1999; Benzing, 1990). Lichens and bryophytes assimilate nutrients primarily from atmospheric deposition on the plant surface (Benzing, 1990; Hietz et al., 1999), whereas ferns and spermatophytes can absorb nutrients from roots as well as their leaves (Reich et al., 2003; Stewart et al., 1995). The leaves of deciduous ferns had lower N and P contents than those of evergreen ferns, although the difference in N concentrations was not significant between the two types of ferns. Lower N and P contents in leaves of deciduous ferns might be the result of the N and P loss through senescent leaf shedding, even though most of N and P were retained efficiently by nutrient resorption (Aerts, 1996; Killingbeck, 1996; Zotz, 2004). In terrestrial plants, leaf N and P contents are lower for evergreen species than for deciduous species (Chen et al., 2013; Güsewell, 2004), although sometimes the contents are lower in deciduous species (Wright et al., 2005). In the ferns and spermatophytes, the leaves had the highest N and P concentrations compared with the stems and roots (Figure 1b). This result indicated that the epiphytes might allocate more of limited nutrients to their leaves for photosynthesis to maximize their growth in the rainy season. These allocations are consistent with those in other epiphytes and terrestrial plants (Zhang et al., 2018; Zotz, 1999). The increased allocation of nutrients to the leaves might be an adaptation to a nutrient‐limited environment (Yan et al., 2016).

The N and P contents were highly correlated across the epiphyte community (Figure 2). The different allometric relationships between N and P also reflected that lichens might need more P than ferns and spermatophytes when they got nutrients from the environment (Figure 2b). In addition, the leaves might need more P than the stems and roots. These different allometric relationships among functional groups and organs are also observed in other terrestrial plants (Kerkhoff et al., 2006; Tian et al., 2018). Furthermore, the scaling exponents between N and P were less than 1 (slopes < 1), which indicated that epiphytes would take up more P than N from the environment. The P content increased faster than the N content (Ågren, 2008; Wright et al., 2004; Zhang et al., 2018), which resulted in decreases in the N:P ratio with increasing leaf nutrient concentration (Elser, Fagan, Kerkhoff, Swenson, & Enquist, 2010). According to the growth rate hypothesis, plants with high growth rates require a high allocation of phosphorus and have low biomass N:P ratios (Sterner & Elser, 2002). Thus, allometric relationships may be useful to successfully predict the relative growth rates of epiphytes with an increase in plant nutrients, as previously confirmed in terrestrial plants (Niklas, 2006; Niklas et al., 2005; Reich et al., 2010). In this study, the scaling exponent (0.61, N ∝ P^0.61^) converted to the coefficient of reduced major axis regression was 0.78, similar to 3/4 power (N ∝ P^3/4^) (Niklas et al., 2005). The conversion formula is a_RMA_ = a_OLS_/*r*, where a_RMA_ is the scaling exponent of reduced major axis regression, a_OLS_ is the slope of the least square regression, and *r* is the correlation coefficient of the least square regression (Niklas, 2006). These conversions did not affect the allometric relationships above. The relatively invariant allometric scaling of N and P might be the result of physiological constraints in the plants (McGroddy, Daufresne, & Hedin, 2004).

### 4.2 Stoichiometry of K and Ca and their roles in the epiphytes

The vascular epiphytes maintained high K concentrations and low N:K ratios in leaves (Table 1, Figure 1), which might be beneficial to their growth in the rainy season. Moreover, the K concentrations in the leaves of ferns and spermatophytes were significantly higher than those in the stems and roots. K is an essential element for plant growth and development (Gajdanowicz et al., 2011; Leigh & Wyn Jones, 1984). High K allocation in the leaves of epiphytes would promote osmoregulation, enzyme activity, and photosynthesis, as previously confirmed in other terrestrial plants (Leigh & Wyn Jones, 1984; Osakabe et al., 2013). However, the growth of epiphytes in the forest canopy is usually stressed by the water supply (Hietz & Wanek, 2003; Laube & Zotz, 2003). One of the important functions of K is alleviating the inhibition of drought stress on growth (Sardans & Peñuelas, 2015). High leaf K concentrations of vascular epiphytes might reduce their leaf water potential and promote the diffusion of water to the leaves (Leigh & Wyn Jones, 1984). Thus, the vascular epiphytes maintained high K concentrations and low N:K ratios in their leaves to acclimatize to drought stress or other environmental stresses in the canopy (Sardans, Peñuelas, et al., 2012). By contrast, the lichens and bryophytes had low K concentrations and high N:K ratios, which are consistent with their very large changes in water content. These two plant types are defined as poikilohydric plants and can survive when the water content is <5%–10% dry weight (Proctor & Tuba, 2002). Furthermore, the leaf growth of ferns and spermatophytes might not be K‐limited on the basis of their low N:K ratios, according to the critical ratios (N:K < 2.1) in wetland vegetation (Olde Venterink, Wassen, Verkroost, & de Ruiter, 2003).

Consistently, significant differences in Ca concentrations and N:Ca ratios among the functional groups or organ types were not observed (Figure 1). The only consistent difference was that the leaves of spermatophytes had the highest Ca concentrations and the lowest N:Ca ratios compared with the other phylogenetic groups. These results indicated that the role of Ca in the epiphytes might not be affected by functional or organ divisions. Ca^2+^ acts as an intracellular secondary messenger and plays an important role in plant defense responses to abiotic or biotic stresses (Bush, 1995; Lecourieux et al., 2006). Furthermore, Ca^2+^ is a crucial regulator of growth and development in plants (Hepler, 2005). A deficiency in calcium will likely reduce growth and adaptation to stress (Reddy, Ali, Celesnik, & Day, 2011), particularly for epiphytes in the unstable forest canopy (Benzing, 1990). The regulation of Ca in epiphytes may be achieved by changing cytosolic Ca^2+^ concentration ([Ca^2+^]_cyt_) when epiphytes respond to developmental signals and environmental stress (Knight & Knight, 2001; White & Broadley, 2003); however, further research remains to explain the conservative stoichiometric differences in epiphytes.

### 4.3 Possible nutritional adaptations in the epiphyte communities

For most epiphyte species in this study, the results are not consistent with the previous expectation that slow growing epiphytes are low in nutrient contents and high in N:P ratios (Laube & Zotz, 2003; Schmidt & Zotz, 2002; Sterner & Elser, 2002). For example, the concentrations of nutrients were higher (N, P, K, and Ca) and the N:P ratios were lower in the leaves of fern species than those in other terrestrial plants (Table 1). Only most of the lichen species showed results consistent with the expectations. Therefore, these results indicated that nutrient constraints in most epiphytes may not be severe, based on the high nutrient concentrations and low N:P ratios that occurred in the rainy season (Table 1). The relatively adequate supply of nutrients in most epiphytes during the optimum growth period might be attributed to a series of adaptive mechanisms, such as efficient resource acquisition and high nutrient retention (Aerts & Chapin, 1999; Winkler & Zotz, 2009). According to the growth rate hypothesis, lichens with relatively low nutrient contents and high N:P ratios might grow slowly (Elser et al., 2000; Sterner & Elser, 2002; Willby, Pulford, & Flowers, 2001), whereas bryophytes, ferns, and spermatophytes might tend to grow relatively rapidly because of their relatively high nutrient concentrations and low N:P ratios (Ågren, 2004; Elser et al., 2003; Nielsen et al., 1996). These assumptions may be contrary to previous studies in which epiphytes are slowly growing plants (Benzing, 1990; Laube & Zotz, 2003; Schmidt & Zotz, 2002), have low nutrient concentrations (Hofstede et al., 1993; Watkins, Rundel, & Cardelús, 2007), and show phosphorus limitation (Zotz, 2004; Zotz & Richter, 2006). These assumptions may also differ from those of stress‐tolerant plants with low contents of nutrient elements, very slow growth, and average relatively high N:P ratios (Aerts & Chapin, 1999; Güsewell, 2004).

To respond to environmental changes, the epiphytes showed stoichiometric plasticity, and to maintain metabolism and growth, they might also have a degree of homeostatic ability. The plasticity of epiphytes was primarily reflected in the wide ranges of element concentrations and ratios across epiphyte species (Table 1, Figure 3). However, the intraspecific variations in element concentrations and ratios were less than the interspecific variations (Table 1). The lower intraspecific plasticity suggested that epiphytes might have a degree of homeostatic ability. The intraspecific variation in epiphytes can also be expressed by the coefficient of variation (CV, CV = (*SD*/mean) × 100%), which is widely used to describe the variability or plasticity in terrestrial plants (Han et al., 2011; McGroddy et al., 2004; Valladares, Sanchez‐Gomez, & Zavala, 2006; Zhang et al., 2018). For example, the CV of N concentrations in *Usnea florida* was 6.02% and much lower than that among species which was 39.36% (Table 1). The trade‐off between flexibility and stability in epiphytes might be an adaptive mechanism in response to changes in N and P supply ratios (Koerselman & Meuleman, 1996). Although terrestrial plants have wide variation in the foliar C:N:P ratio and lower homeostasis than animals or bacteria (Elser et al., 2010; Güsewell, 2004), a degree of stoichiometric homeostasis remains (Elser et al., 2010). The epiphytes with homeostatic ability have the capacity to adjust their C:N:P stoichiometry to an optimal value by different mechanisms (Sardans, Rivas‐Ubach, & Peñuelas, 2012), such as fundamental physiological constraints (McGroddy et al., 2004). Moreover, the stoichiometric homoeostasis of epiphytes can also be evaluated with increased accuracy by homeostatic coefficients (*H*) through fertilizer experiments (Sterner & Elser, 2002; Yu et al., 2011).

## 5 CONCLUSIONS

In this study, we investigated the ecological stoichiometry of dominant epiphytes to reveal the nutrient status and possible adaptations of the epiphyte community in a subtropical forest canopy. We found that the element contents and ratios of epiphytes at the species level were highly variable. Moreover, these stoichiometric characteristics of epiphytes differed significantly among functional groups. Compared with terrestrial plants, most epiphytes maintained high nutrient contents during the rainy season. High nutrient contents and various stoichiometric characteristics indicated that epiphytes in the forest canopy might have evolved their own nutritional adaptations, such as high nutrient allocations to the leaves in vascular epiphytes, lower potassium contents in poikilohydric epiphytes, and the trade‐off between stoichiometric plasticity and homeostasis. However, more research is still needed to reveal the potential mechanism of the epiphyte communities.

## CONFLICT OF INTEREST

None declared.

## AUTHOR CONTRIBUTIONS

Jun‐Biao Huang and Wen‐Yao Liu designed the experiment; Jun‐Biao Huang collected the experimental data; Su Li, Liang Song, Hua‐Zheng Lu, Xian‐Meng Shi, Xi Chen, Tao Hu, Shuai Liu, Tao Liu assisted in the experiment and chemical analysis; Jun‐Biao Huang analyzed the experimental data and wrote the first draft of the manuscript; Wen‐Yao Liu contributed substantially to manuscript revisions.

## Data Availability

Data associated with this work are available in the Dryad Digital Repository: https://doi.org/10.5061/dryad.cz8w9ghzv.
